# Age-dependent association between lifestyle oxidative balance score and bone mineral density in children and adolescents: evidence from the NHANES 2015–2018

**DOI:** 10.3389/fphys.2025.1618996

**Published:** 2025-07-04

**Authors:** Suman Li, Yuan Mao

**Affiliations:** ^1^ Department of Pediatrics, Yuyao Hospital of Traditional Chinese Medicine, Ningbo, Zhejiang, China; ^2^ Department of Rehabilitation Medicine, Yuyao Hospital of Traditional Chinese Medicine, Ningbo, Zhejiang, China

**Keywords:** oxidative balance score, bone mineral density, children, adolescents, national health and nutrition examination survey

## Abstract

**Background:**

Oxidative balance score (OBS) integrates pro- and antioxidant exposures, potentially influencing skeletal health. This study aimed to examine the association between OBS with bone mineral density (BMD) in children and adolescents.

**Methods:**

Individuals aged ≤18 years from the National Health and Nutrition Examination Survey were included. The OBS was determined based on four lifestyle factors and sixteen dietary nutrients. Baseline characteristics were compared by gender. Generalized linear regression models were utilized to assess the relationships between total, lifestyle, and dietary OBS with lumbar spine, pelvis, and total BMD. Interaction and subgroup analyses were used to examine age-related modifications. The mediation effect of oxidative stress-related indicators in the relationship between OBS and BMD was analyzed using mediation analysis.

**Results:**

Finally, 1196 children and adolescents with a median age of 13 years were included. Lifestyle OBS showed a significant positive correlation with all BMD measures. However, the association reversed to a negative correlation after adjusting for confounders. Sensitivity analysis identified age as a key modifier. Subgroup analysis revealed that lifestyle OBS positively correlated with lumbar spine and total BMD in younger individuals (≤13 years) but negatively correlated with all BMD measures in older participants (>13 years). A nonlinear relationship between lifestyle OBS and BMD was observed. Higher lifestyle OBS was associated with greater physical activity and lower cotinine levels. Additionally, uric acid and GGT were the potential mediators between lifestyle OBS and BMD.

**Conclusion:**

Lifestyle OBS exhibits an age-dependent association with BMD. These findings highlight the importance of age considerations in lifestyle-BMD research and potential implications for bone health strategies in youth.

## 1 Introduction

Bone mineral density (BMD) is a key determinant of skeletal health, particularly during the periods of rapid bone growth and mineralization in childhood and adolescence (Gusmao et al., 2023). During these developmental stages, optimal peak bone mass (PBM) accumulation is crucial for reducing the risk of osteoporosis and fractures later in life (Deng et al., 2021). Studies have shown that a 5% increase in PBM throughout childhood and adolescence can reduce the risk of osteoporotic fractures by 40%, while a 10% increase can halve the risk (Ouyang et al., 2022). Numerous factors influence BMD, including genetics, physical activity (PA), hormonal regulation, and dietary intake (Ahn and Yoo, 2023). Evidence suggests a potential causal relationship between diet-derived pro-oxidants and antioxidants and bone health (Yuan et al., 2024). Oxidative stress, caused by an imbalance between reactive oxygen species (ROS) production and antioxidant defense, has been associated with increased bone resorption and reduced bone formation (Zhang et al., 2023). Reducing oxidative stress has been shown to protect bone mass in lipopolysaccharide-treated rats (Tao and Ma, 2024).

The oxidative balance score (OBS) is a composite index calculated based on 20 different dietary and lifestyle components of antioxidants and pro-oxidants, used to assess an individual’s oxidative balance status (Liu et al., 2024; Xu Z. et al., 2024). Generally, a higher OBS indicates greater antioxidant capacity and lower oxidative stress (Zhang et al., 2022). Several dietary components, such as vitamin and polyunsaturated fatty acids, enhance antioxidant defense, whereas high-fat and high-sugar diets contribute to oxidative stress (Wang et al., 2023). Additionally, lifestyle factors, including smoking and PA, further modulate oxidative balance (Zhou and Han, 2024). Recent studies have further demonstrated that OBS is significantly associated with several oxidative stress biomarkers, such as serum albumin and uric acid (Zhu et al., 2025; Chang et al., 2024; Song et al., 2023). This evidence supports the validity of OBS as an effective measure for evaluating an individual’s oxidative/antioxidative balance. While OBS is associated with various chronic diseases, including cardiovascular disease (Chen et al., 2024), diabetes (Wu et al., 2023), and cancer (Hasani et al., 2023), its role in bone health, particularly in children and adolescents, remains underexplored.

The National Health and Nutrition Examination Survey (NHANES) includes comprehensive dietary assessments, biomarkers of oxidative stress, and BMD measurements obtained via dual-energy X-ray absorptiometry (DXA), providing a robust dataset for investigating the relationship between oxidative balance and bone health. This study aimed to explore the association between oxidative balance and BMD in children and adolescents using NHANES 2015–2018 data. Additionally, it further evaluated the potential mediation effects of oxidative stress-related biomarkers in this relationship. Through these analyses, this study tried to determine whether maintaining a favorable oxidative balance through diet and lifestyle contributes to improved skeletal health in young populations, potentially informing early prevention strategies for bone-related diseases.

## 2 Methods

### 2.1 Study design and population and sample size calculation

This study utilized data from NHANES 2015–2018. NHANES is a nationally representative survey of the civilian, non-institutionalized U.S. population, conducted by the National Center for Health Statistics (NCHS), using a complex, multistage, probability sampling design. The survey integrates interviews, physical examinations, and laboratory tests to assess the health and nutritional status of participants. The NHANES data were approved by the NCHS Research Ethics Review Board, and written informed consent was obtained (Song et al., 2024). We adhered to the research data usage guidelines to ensure that the data were used solely for statistical analysis. Since all downloaded data were de-identified, ethical approval was not required.

Among the initial 18,824 samples, individuals aged ≤18 years were included (n = 7261). The following exclusion criteria were applied to the remaining population: (1) lack of lumbar spine BMD, pelvis BMD, and total BMD data (n = 4297), (2) lack of information on the 16 dietary OBS components (n = 711), and (3) lack of information on the four lifestyle OBS components (n = 1057). Ultimately, 1196 participants were included in this study.

Subsequently, the sample size was calculated using G*Power 3.1.3. The effect size was set to a medium level (effect size = 0.5), with a significance level (α) of 0.05 and a statistical power of 95%. The minimum required sample size for a two-tailed t-test was calculated to be 220 participants. A total of 1196 participants were ultimately included in this study, meeting the required standard.

### 2.2 BMD testing

All participants underwent BMD testing via DXA, conducted by certified radiology technicians using a Hologic QDR-4500A fan-beam densitometer (Hologic; MA, United States). DXA scan data were analyzed using Hologic APEX software (version 4.0) (Tang et al., 2022). In this study, lumbar spine BMD, pelvis BMD, and total BMD were used as outcome variables.

### 2.3 OBS determination

OBS was determined by integrating 16 dietary factors and four lifestyle components (Liu et al., 2024). The dietary OBS components included dietary fiber, total fat, carotene, riboflavin, niacin, total folate, vitamins B6/B12/C/E, calcium, copper, iron, magnesium, selenium, and zinc. These variables were obtained from the NHANES 24 h dietary recall interviews. The lifestyle OBS components consisted of body mass index (BMI), PA, alcohol consumption, and smoking status. BMI was calculated as weight in kilograms divided by height in meters squared. PA was quantified in metabolic equivalent tasks (MET) and calculated based on the cumulative time spent in transportation and moderate-intensity activities per week (Tian et al., 2022). MET score = weekly frequency × duration of each PA × assigned MET value per activity (Xu W. et al., 2024). Smoking exposure was assessed using serum cotinine levels. All OBS components were treated as continuous variables. Among them, total fat, iron, BMI, alcohol consumption, and smoking status were considered pro-oxidants, while the remaining variables were classified as antioxidants.

The OBS score was calculated as previously proposed (Zhang et al., 2022). Alcohol consumption was categorized into three groups: heavy drinkers (≥15 g/day for females, ≥30 g/day for males), non-heavy drinkers (0–15 g/day for females, 0–30 g/day for males), and non-drinkers, who were assigned scores of 0, 1, and 2, respectively. The remaining components were categorized into gender-specific tertiles. For antioxidant components, participants in the lowest to highest tertiles received scores of 0, 1, and 2, respectively, whereas pro-oxidant components were scored in the reverse order. The total OBS was calculated as the sum of the scores for all individual components (Supplementary Table S1).

### 2.4 Covariates

Covariates included demographic characteristics such as age, gender, race, education, and family poverty income ratio (PIR). Demographic data were collected through standardized household interviews. Race was categorized as Hispanic and non-Hispanic. Education was classified as ≥9th grade and <9th grade. Our study also included stimulant use, encompassing amphetamine, methylphenidate, dextroamphetamine, dexmethylphenidate, and other unspecified central nervous system stimulants. Participants were asked about prescription medication use in the past 30 days. The questions included: “Was any prescription medication taken in the past 30 days?” “Medication name,” “How long was medication taken?” and “What is the main reason for which you use medication?” Participants aged over 16 years answered the questions themselves, while younger participants had their responses provided by a proxy (Fu et al., 2022).

### 2.5 Oxidative stress indicators

Based on the potential association between OBS and oxidative stress, this study also included several oxidative stress-related indicators, including serum albumin, serum 25(OH)D, uric acid, and gamma-glutamyl transpeptidase (GGT). Serum albumin, uric acid, and GGT concentrations were measured using the Beckman Coulter UniCel DxC800 system, while serum 25(OH)D was quantified using ultra-performance liquid chromatography–tandem mass spectrometry (UPLC-MS/MS). Detailed measurement procedures are publicly available at https://wwwn.cdc.gov/Nchs/Nhanes/continuousnhanes (Song et al., 2023).

### 2.6 Statistical analysis

Data analysis was conducted using SPSS software version 26, with statistical significance set at p<0.05. Categorical variables were expressed as percentages [n (%)] and compared using the chi-square test. Normally distributed continuous variables were presented as mean ± standard deviation and compared using the t-test. Non-normally distributed continuous variables were presented as median [interquartile range (IQR)] and compared using the Mann-Whitney U test or the Kruskal–Wallis test.

A univariate generalized linear regression analysis was performed to examine the associations of OBS (lifestyle OBS, dietary OBS, and total OBS) with BMD (lumbar spine BMD, pelvis BMD, and total BMD). Further multivariate generalized linear regression analysis was conducted to explore the relationship between lifestyle OBS and the three BMD measures, with covariate adjustment, including age, gender, race, education, family PIR, and stimulant use. Sensitivity analysis was performed using a stepwise variable exclusion method. Based on the median values of lifestyle OBS and age, all participants were classified into four groups: low age + low lifestyle OBS, low age + high lifestyle OBS, high age + low lifestyle OBS, and high age + high lifestyle OBS. Differences in the three BMD measures were compared across these combined indicator groups. The interaction effect of age on the association between lifestyle OBS and the three BMD measures was evaluated, and both univariate and multivariate generalized linear regression analyses were conducted within age subgroups. The dose-response relationship between lifestyle OBS and BMD was analyzed using the generalized additive model (GAM) analysis (*family*: Gaussian; *link function*: identity). Based on the nonlinear patterns identified by GAM, threshold effect analysis was further performed to determine the inflection points in the associations between lifestyle OBS and BMD. Participants were grouped based on the median value of lifestyle OBS, and differences in lifestyle OBS-related variables, demographic characteristics, and stimulant use were analyzed between the two groups. Stratified analyses were subsequently performed to explore the potential effect modification by gender on the association between lifestyle OBS and BMD. Finally, mediation analysis was conducted to investigate the potential mechanisms underlying the relationship between lifestyle OBS and BMD mediated by oxidative stress indicators.

## 3 Results

### 3.1 Baseline characteristics

This study enrolled 1196 participants, comprising 617 males (51.589%) and 579 females (48.411%), with a median age of 13 years. Baseline characteristics stratified by gender and statistical comparisons are presented in Table 1. The race composition included 64.130% non-Hispanic individuals (406 males, 361 females) and 35.870% Hispanic individuals (211 males, 218 females), with no significant gender differences (p = 0.213). Educational attainment was comparable between genders, with 33.6% of participants having completed education beyond ninth grade. Household socioeconomic status, assessed by the PIR, demonstrated a median value of 1.580 across the cohort, with no gender disparity (p = 0.458). Totally, 34 participants (2.843%) reported stimulant use, with males exhibiting significantly higher prevalence (27 cases) than females (p<0.001). All oxidative stress-related indicators, including serum albumin, serum 25(OH)D, uric acid, and GGT, were significantly lower in females than in males (p<0.01). Among lifestyle OBS-related variables, alcohol consumption was absent in all participants, and BMI showed a median value of 21.4 kg/m^2^, neither displaying gender differences (p>0.05). Cotinine levels and physical activity demonstrated median values of 0.030 ng/mL and 360.0 min/week, respectively, both significantly elevated in males compared to females (p<0.01). There were significant gender differences in 14 of 16 dietary OBS-related variables, with males exhibiting higher values than females (p<0.001), except for carotene and vitamin C. The mean lifestyle OBS was 1.292, while median dietary OBS and total OBS were 14.0 and 19.0, respectively, all showing significant gender variations (p<0.01). BMD assessments yielded median values of 0.870 g/cm^2^ (lumbar spine), 1.060 g/cm^2^ (pelvis), and 0.950 g/cm^2^ (total). Notably, males demonstrated significantly lower lumbar spine BMD compared to females (p<0.001), while the other two BMD measures showed no gender disparity.

**TABLE 1:** Baseline characteristics of the study populations.

Variables	Total (n = 1196)	Male (n = 617)	Female (n = 579)	p
Age (year), median [IQR]	13.000 [10.000,16.000]	13.000 [10.000,16.000]	13.000 [10.000,16.000]	0.915
Race, n (%)				
Non-Hispanic	767 (64.130)	406 (65.802)	361 (62.349)	0.213
Hispanic	429 (35.870)	211 (34.198)	218 (37.651)	
Education, n (%)				
<9th grade	794 (66.388)	423 (68.558)	371 (64.076)	0.101
≥9th grade	402 (33.612)	194 (31.442)	208 (35.924)	
Family PIR, median [IQR]	1.580 [0.900, 2.880]	1.640 [0.920, 2.880]	1.540 [0.850, 2.880]	0.458
Stimulant use, n (%)				
No	1162 (97.157)	590 (95.624)	572 (98.791)	<0.001
Yes	34 (2.843)	27 (4.376)	7 (1.209)	
Serum albumin (g/dL), median [IQR]	4.500 [4.300,4.700]	4.600 [4.400,4.800]	4.400 [4.200,4.600]	<0.001
Serum 25(OH)D (nmol/L), median [IQR]	57.000 [45.700,68.500]	58.800 [47.400,69.000]	55.000 [42.900,67.300]	0.005
Uric acid (mg/dL), median [IQR]	4.900 [4.100,5.800]	5.600 [4.800,6.400]	4.300 [3.700,5.000]	<0.001
GGT (IU/L), median [IQR]	13.000 [11.000,17.000]	14.000 [12.000,18.000]	12.000 [10.000,14.000]	<0.001
BMI (kg/m^2^), median [IQR]	21.400 [18.300, 25.700]	21.200 [18.200, 25.100]	21.700 [18.500, 26.200]	0.162
Cotinine (ng/mL), median [IQR]	0.030 [0.010, 0.120]	0.030 [0.010, 0.170]	0.020 [0.010, 0.090]	0.004
Alcohol (g/d), median [IQR]	0.000 [0.000, 0.000]	0.000 [0.000, 0.000]	0.000 [0.000, 0.000]	0.343
PA (MET-min/week), median [IQR]	360.000 [0.000, 2,800.000]	720.000 [0.000, 3760.000]	160.000 [0.000, 1760.000]	<0.001
Dietary fiber (g/d), median [IQR]	13.700 [9.950, 18.300]	14.550 [10.500, 19.200]	13.000 [9.450, 17.000]	<0.001
Total fat (g/d), median [IQR]	70.460 [54.315, 93.130]	74.740 [58.425, 98.890]	65.600 [50.965, 86.380]	<0.001
Carotene (RE/d), median [IQR]	49.708 [24.583, 128.813]	50.375 [25.688, 143.625]	48.917 [23.729, 114.333]	0.239
Riboflavin (mg/d), median [IQR]	1.811 [1.289, 2.358]	1.923 [1.424, 2.622]	1.695 [1.162, 2.173]	<0.001
Niacin (mg/d), median [IQR]	21.138 [16.005, 27.976]	23.943 [17.407, 31.088]	19.395 [14.682, 25.308]	<0.001
Vitamin B6 (mg/d), median [IQR]	1.646 [1.198, 2.218]	1.782 [1.303, 2.489]	1.505 [1.107, 1.957]	<0.001
Total folate (mcg/d), median [IQR]	342.500 [248.500, 476.500]	375.000 [277.500, 512.000]	312.500 [217.500, 430.000]	<0.001
Vitamin B12 (mcg/d), median [IQR]	4.220 [2.615, 6.030]	4.805 [3.165, 6.825]	3.700 [2.320, 5.305]	<0.001
Vitamin C (mg/d), median [IQR]	56.700 [28.000, 98.350]	58.600 [30.050, 105.250]	53.450 [25.950, 93.950]	0.114
Vitamin E (ATE) (mg/d), median [IQR]	6.660 [4.825, 9.155]	7.075 [4.980, 9.695]	6.325 [4.490, 8.505]	<0.001
Calcium (mg/d), median [IQR]	874.500 [635.500, 1204.500]	935.500 [685.000, 1327.000]	809.500 [590.000, 1116.500]	<0.001
Magnesium (mg/d), median [IQR]	224.500 [173.000, 285.000]	236.500 [184.000, 304.500]	211.000 [162.500, 271.000]	<0.001
Iron (mg/d), median [IQR]	13.195 [9.765, 17.920]	14.500 [10.900, 19.390]	11.785 [8.455, 16.305]	<0.001
Zinc (mg/d), median [IQR]	9.405 [7.005, 12.685]	10.485 [7.850, 14.200]	8.420 [6.290, 11.125]	<0.001
Copper (mg/d), median [IQR]	0.868 [0.661, 1.110]	0.909 [0.701, 1.216]	0.815 [0.621, 1.039]	<0.001
Selenium (mcg/d), median [IQR]	95.050 [72.600, 124.800]	105.900 [78.550, 141.200]	87.550 [68.050, 111.650]	<0.001
Lifestyle OBS, mean (±SD)	1.292 (±0.455)	1.331 (±0.470)	1.250 (±0.433)	0.002
Dietary OBS, median [IQR]	14.000 [9.000, 19.000]	13.000 [9.000, 19.000]	16.000 [10.000, 20.000]	<0.001
Total OBS, median [IQR]	19.000 [14.000, 25.000]	18.000 [13.000, 24.000]	20.000 [15.000, 25.000]	<0.001
Lumbar spine BMD (g/cm^2^), median [IQR]	0.870 [0.700, 1.010]	0.820 [0.680, 0.990]	0.910 [0.730, 1.040]	<0.001
Pelvis BMD (g/cm^2^), median [IQR]	1.060 [0.870, 1.220]	1.020 [0.860, 1.220]	1.080 [0.890, 1.220]	0.062
Total BMD (g/cm^2^), median [IQR]	0.950 [0.810, 1.070]	0.940 [0.820, 1.070]	0.950 [0.810, 1.060]	0.324

OBS, oxidative balance score; PIR, poverty income ratio; GGT, gamma-glutamyl transpeptidase; BMI, body mass index; PA, physical activity; MET, metabolic equivalent tasks; RE, retinol equivalent; ATE, alpha-tocopherol equivalent; BMD, bone mineral density; IQR, interquartile range.

### 3.2 Association of OBS with BMD

Subsequently, the relationship between OBS and BMD was investigated using generalized linear regression analysis. As shown in Table 2, neither total OBS nor dietary OBS exhibited significant associations with lumbar spine BMD, pelvis BMD, or total BMD (p > 0.05). However, lifestyle OBS demonstrated significant positive correlations with all three BMD measures (p < 0.05), with regression coefficients (95% confidence intervals) of 0.019 (0.011, 0.028) for lumbar spine BMD, 0.012 (0.002, 0.022) for pelvis BMD, and 0.015 (0.008, 0.022) for total BMD.

**TABLE 2:** Association of OBS with BMD.

Variables	Lumbar spine BMD		Pelvis BMD		Total BMD	
	β (95%CI)	p	β (95%CI)	p	β (95%CI)	p
Total OBS	0.001 (−0.001, 0.002)	0.492	0.000 (−0.002, 0.002)	0.942	0.000 (−0.001, 0.001)	0.909
Lifestyle OBS	0.019 (0.011, 0.028)	<0.001	0.012 (0.002, 0.022)	0.020	0.015 (0.008, 0.022)	<0.001
Dietary OBS	0.000 (−0.002, 0.002)	0.813	−0.001 (−0.003, 0.002)	0.572	−0.001 (−0.002, 0.001)	0.432

OBS, oxidative balance score; BMD, bone mineral density; CI, confidence interval.

To further explore the association between lifestyle OBS and BMD, adjustments were made for potential confounding factors including age, gender, race, education, family PIR, and stimulant use. Post-adjustment results (Table 3) revealed that while lifestyle OBS remained significantly associated with all three BMD measures (p < 0.05), the association reversed to negative correlations (β < 0). This suggested that confounding factors may influence the observed relationship between lifestyle OBS and BMD.

**TABLE 3:** Association of lifestyle OBS with BMD.

Variables	Lumbar spine BMD		Pelvis BMD		Total BMD	
	β (95%CI)	p	β (95%CI)	p	β (95%CI)	p
Lifestyle OBS	−0.007 (−0.013, −0.001)	0.022	−0.026 (−0.033, −0.018)	<0.001	−0.010 (−0.015, −0.005)	<0.001

Adjusted for age, gender, race, education, family poverty income ratio, and stimulant use.

OBS, oxidative balance score; BMD, bone mineral density; CI, confidence interval.

### 3.3 Association between lifestyle OBS and BMD was affected by age

To investigate the reversal of associations between lifestyle OBS and BMD before and after confounder adjustment, sensitivity analyses were conducted using the stepwise variable exclusion approach. As shown in Table 4, lifestyle OBS remained significantly negatively associated with BMD after individually excluding gender, race, education, family PIR, and stimulant use (p < 0.05). However, the association reverted to positive correlations when age was excluded, indicating that age may influence the observed relationship.

**TABLE 4:** Sensitivity analysis between lifestyle OBS and BMD.

Excluded variables	Lumbar spine BMD		Pelvis BMD		Total BMD	
	β (95%CI)	p	β (95%CI)	p	β (95%CI)	p
Age	0.014 (0.006, 0.021)	<0.001	0.002 (−0.007, 0.012)	0.657	0.008 (0.002, 0.014)	0.010
Gender	−0.009 (−0.015, −0.003)	0.002	−0.026 (−0.034, −0.019)	<0.001	−0.009 (−0.014, −0.005)	<0.001
Race	−0.008 (−0.014, −0.002)	0.011	−0.026 (−0.034, −0.019)	<0.001	−0.011 (−0.016, −0.006)	<0.001
Education	−0.006 (−0.012, −0.001)	0.042	−0.023 (−0.030, −0.015)	<0.001	−0.009 (−0.014, −0.004)	<0.001
Family PIR	−0.005 (−0.011, 0.001)	0.057	−0.023 (−0.030, −0.016)	<0.001	−0.009 (−0.014, −0.005)	<0.001
Stimulant use	−0.007 (−0.013, −0.001)	0.025	−0.025 (−0.033, −0.018)	<0.001	−0.010 (−0.015, −0.005)	<0.001

OBS, oxidative balance score; BMD, bone mineral density; CI, confidence interval; PIR, poverty income ratio.

**FIGURE 1:**
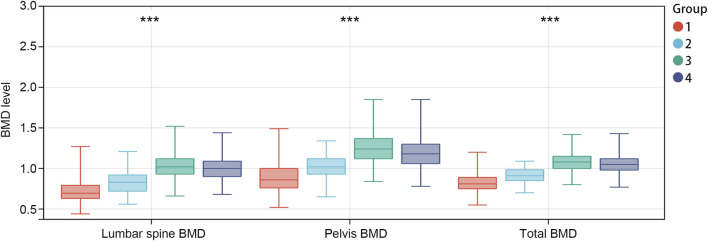
BMD levels in different combined indicator subgroups. Group 1: low age + low lifestyle OBS, group 2: low age + high lifestyle OBS, group 3: high age + low lifestyle OBS, group 4: high age + high lifestyle OBS. ***p<0.001. OBS, OBS: oxidative balance score; BMD: bone mineral density.

Participants were subsequently stratified into four groups based on median age and lifestyle OBS: low age + low lifestyle OBS, low age + high lifestyle OBS, high age + low lifestyle OBS, and high age + high lifestyle OBS. Analysis of the combined indicators (Figure 1) revealed comparable BMD levels in both high-age subgroups (high age + low/high lifestyle OBS, >13 years), which exceeded those in low-age subgroups. Within the low-age groups (≤13 years), the high lifestyle OBS subgroup exhibited higher BMD compared to the low lifestyle OBS subgroup. These findings suggest that lifestyle OBS primarily exerts a beneficial effect on BMD enhancement in younger individuals (≤13 years).

Interaction and subgroup analyses were performed to further explore age-dependent effects (Table 5). Significant interaction effects were observed between age and lifestyle OBS across all BMD measures (p for interaction <0.001), regardless of confounder adjustment. Subgroup analysis stratified by median age (≤13 vs. >13 years) demonstrated divergent associations. In the low age group, lifestyle OBS positively correlated with lumbar spine BMD (β = 0.024, 95% CI: 0.013–0.035) and total BMD (β = 0.017, 95% CI: 0.006–0.028), but showed no significant association with pelvis BMD (p = 0.102). In the high age group (>13 years), significant negative correlations emerged between lifestyle OBS and all BMD measures: lumbar spine (β = −0.015, 95% CI: 0.024–0.006), pelvis (β = −0.011, 95% CI: 0.020–0.002), and total BMD (β = −0.013, 95% CI: 0.021–0.005) (all p<0.05).

**TABLE 5:** Interaction and subgroup analysis.

Methods	Subgroup	Lumbar spine BMD			Pelvis BMD			Total BMD		
		β (95%CI)	p	p for interaction	β (95%CI)	p	p for interaction	β (95%CI)	p	p for interaction
Univariate	Age			<0.001			<0.001			<0.001
	Low	0.011 (0.002, 0.020)	0.013		0.006 (−0.005, 0.018)	0.293		0.008 (0.000, 0.015)	0.038	
	High	−0.012 (−0.021, −0.004)	0.003		−0.028 (−0.038, −0.018)	<0.001		−0.011 (−0.018, −0.005)	0.001	
Multivariate	Age			<0.001			<0.001			<0.001
	Low	0.019 (0.010, 0.028)	<0.001		0.011 (−0.002, 0.024)	0.085		0.011 (0.003, 0.019)	0.007	
	High	−0.011 (−0.020, −0.003)	0.010		−0.031 (−0.042, −0.020)	<0.001		−0.013 (−0.019, −0.006)	<0.001	

Univariate method is unadjusted, and multivariate method is adjusted for family poverty income ratio, gender, race, education, and stimulant use.

OBS, oxidative balance score; BMD, bone mineral density; CI, confidence interval.

### 3.4 Nonlinear relationships between lifestyle OBS and BMD

**FIGURE 2:**
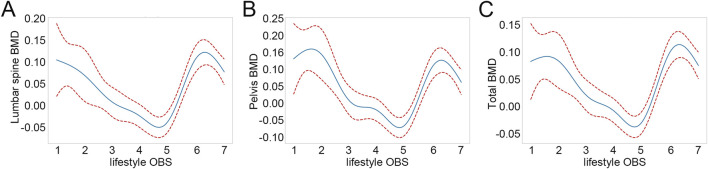
Nonlinear relationships between lifestyle OBS and BMD. **(A)** Nonlinear relationships between lifestyle OBS and lumbar spine BMD. **(B)** Nonlinear relationships between lifestyle OBS and pelvis BMD. **(C)** Nonlinear relationships between lifestyle OBS and total BMD. OBS: oxidative balance score; BMD: bone mineral density.

To elucidate the dose-response relationships between lifestyle OBS and BMD, GAM analysis was employed. As illustrated in Figures 2A–C, lumbar spine BMD, pelvis BMD, and total BMD initially decreased and subsequently increased with rising lifestyle OBS. Subsequently, a threshold effect analysis was conducted to further investigate the U-shaped associations between lifestyle OBS and the three types of BMD. As shown in Supplementary Table S2, a nonlinear relationship was observed between lifestyle OBS and lumbar spine BMD, pelvis BMD, and total BMD (all LRT p<0.001), with corresponding inflection points at 4.559, 4.712, and 4.681, respectively. For lumbar spine BMD, a significant negative association was observed when lifestyle OBS <4.559 (β = −0.043, 95% CI: 0.060, −0.025, p<0.001), whereas the association significantly reversed to positive when lifestyle OBS ≥4.559 (β = 0.066, 95% CI: 0.051, 0.080, p<0.001). Similarly, a significant negative association was found between lifestyle OBS and pelvis BMD when OBS <4.712 (β = −0.057, 95% CI: 0.077, −0.037, p<0.001), and a significant positive association when OBS ≥4.712 (β = 0.074, 95% CI: 0.055, 0.092, p<0.001). A comparable pattern was observed for total BMD, where a significant negative association was present when lifestyle OBS <4.681 (β = −0.035, 95% CI: 0.049, −0.022, p<0.001), and a significant positive association when lifestyle OBS ≥4.681 (β = 0.059, 95% CI: 0.046, 0.070, p<0.001).

The inflection point for BMD trend reversal occurred at a lifestyle OBS of nearly 5, which is also the median value of lifestyle OBS. Therefore, we further examined the differences in demographic factors and lifestyle OBS-related variables between different OBS groups based on this median value. Regarding lifestyle OBS-related variables, cotinine levels and MET were significantly different between the two groups (p<0.001), whereas BMI and alcohol consumption showed no significant difference (Table 6). The high lifestyle OBS group had lower cotinine levels and higher MET levels. Notably, in the low lifestyle OBS group, only 13.459% engaged in active PA (MET-min-week ≥2,880), whereas in the high lifestyle OBS group, the proportion was 47.278%. In terms of demographic characteristics, individuals in the high lifestyle OBS group were older (p<0.001). Additionally, gender, education, and family PIR were significantly different between the high and low lifestyle OBS groups (p<0.01). We also compared oxidative stress-related indicators between the high and low lifestyle OBS groups. Although the median serum albumin level was 4.500 g/dL in both groups, the difference was statistically significant (p<0.001). Compared with the low lifestyle OBS group, the high lifestyle OBS group had significantly lower levels of uric acid and GGT (p<0.01). However, no significant difference was observed in serum 25(OH)D levels between the two groups (p = 0.281). These findings suggest that a higher lifestyle OBS may be associated with a more favorable oxidative stress status.

**TABLE 6:** Differences of variables between low and high lifestyle OBS groups.

Variables	Total (n = 1196)	Lifestyle OBS≤5 (n = 847)	Lifestyle OBS>5 (n = 847)	p
BMI (kg/m^2^), median [IQR]	21.400 [18.300,25.700]	21.500 [17.800,27.400]	21.200 [19.500,23.700]	0.245
Cotinine (ng/mL), median [IQR]	0.030 [0.010,0.120]	0.040 [0.010,0.250]	0.020 [0.010,0.040]	<0.001
Alcohol (g/d), median [IQR]	0.000 [0.000,0.000]	0.000 [0.000,0.000]	0.000 [0.000,0.000]	0.898
MET-total, median [IQR]	360.000 [0.000,2800.000]	0.000 [0.000,720.000]	2,880.000 [1520.000,5280.000]	<0.001
MET-inactive, median [IQR]	917 (76.672)	733 (86.541)	184 (52.722)	<0.001
MET-active, median [IQR]	279 (23.328)	114 (13.459)	165 (47.278)	
Age (year), median [IQR]	13.000 [10.000,16.000]	11.000 [9.000,16.000]	15.000 [13.000,17.000]	<0.001
Gender, n (%)				
Male	617 (51.589)	413 (48.760)	204 (58.453)	0.002
Female	579 (48.411)	434 (51.240)	145 (41.547)	
Race, n (%)				
Non-Hispanic	767 (64.130)	532 (62.810)	235 (67.335)	0.138
Hispanic	429 (35.870)	315 (37.190)	114 (32.665)	
Education, n (%)				
<9th grade	794 (66.388)	608 (71.783)	186 (53.295)	<0.001
≥9th grade	402 (33.612)	239 (28.217)	163 (46.705)	
Family PIR, median [IQR]	1.580 [0.900,2.880]	1.490 [0.830,2.600]	1.860 [1.090,3.680]	<0.001
Stimulant use, n (%)				
No	1162 (97.157)	821 (96.930)	341 (97.708)	0.462
Yes	34 (2.843)	26 (3.070)	8 (2.292)	
Serum albumin (g/dL), median [IQR]	4.500 [4.300,4.700]	4.500 [4.300,4.700]	4.500 [4.400,4.700]	<0.001
Serum 25(OH)D (nmol/L), median [IQR]	57.000 [45.700,68.500]	56.600 [45.500,67.900]	57.400 [45.900,69.300]	0.281
Uric acid (mg/dL), median [IQR]	4.900 [4.100,5.800]	5.100 [4.200,6.100]	4.800 [4.000,5.600]	0.002
GGT (IU/L), median [IQR]	13.000 [11.000,17.000]	14.000 [11.000,18.000]	12.000 [11.000,15.000]	<0.001

OBS, oxidative balance score; PIR, poverty income ratio; BMI, body mass index; MET, metabolic equivalent tasks; BMD, bone mineral density; GGT, gamma-glutamyl transpeptidase; IQR, interquartile range.

Furthermore, to investigate whether the association between lifestyle OBS and BMD varies by gender, an interaction term between lifestyle OBS group and gender was included in the regression model. As presented in Table 7, although lifestyle OBS was significantly associated with increased BMD across all sites, the interaction effect between lifestyle OBS and gender was not statistically significant (p>0.05). This suggests that the beneficial association of lifestyle OBS with BMD does not differ substantially between males and females.

**TABLE 7:** Association between lifestyle OBS categories and BMD with gender-stratified interactions.

Parameters	Lumbar spine BMD		Pelvis BMD		Total BMD	
	β (95%CI)	p	β (95%CI)	p	β (95%CI)	p
lifestyle OBS-high	0.116 (0.084, 0.147)	<0.001	0.127 (0.088, 0.166	<0.001	0.097 (0.071, 0.123)	<0.001
lifestyle OBS-low	0.000^a^		0.000^a^			
female	0.062 (0.037, 0.087)	<0.001	0.032 (4.16E-05, 0.063)	0.050	−0.010 (−0.028, 0.014)	0.513
male	0.000^a^		0.000^a^			
lifestyle OBS-high × female	0.010 (−0.037, 0.056)	0.682	−0.020 (−0.079, 0.039)	0.500	−0.000 (−0.042, 0.036)	0.873
lifestyle OBS-high × male	0.000^a^		0.000^a^			
lifestyle OBS-low × female	0.000^a^		0.000^a^			
lifestyle OBS-low × male	0.000^a^		0.000^a^			
scale	0.034^b^ (0.032, 0.037)		0.550^b^ (0.050, 0.059)		0.024^b^ (0.022, 0.026)	

^a^ Parameter set to zero due to redundancy.

^b^ Estimated by Maximum Likelihood Estimation.

OBS, oxidative balance score; BMD, bone mineral density; CI, confidence interval.

### 3.5 Mediation effects of oxidative stress-related indicators in the association between lifestyle OBS and BMD

**FIGURE 3:**
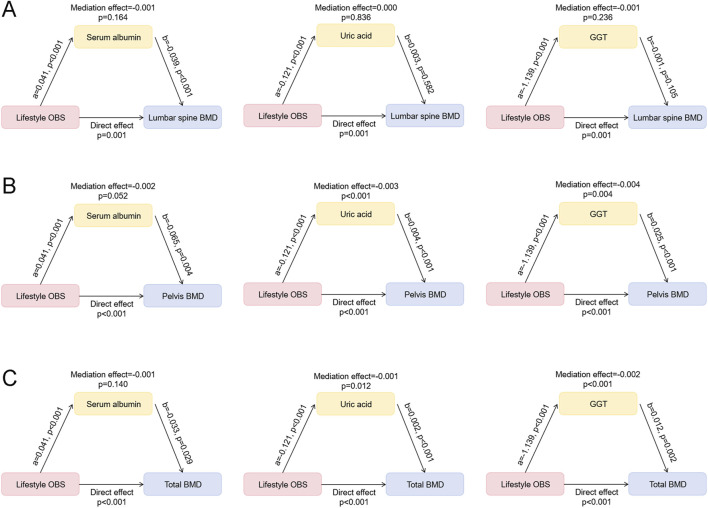
Mediation effects of oxidative stress-related indicators in the association between lifestyle OBS and BMD. **(A)** Mediation effects of serum albumin, uric acid, and GGT in the association between lifestyle OBS and lumbar spine BMD. **(B)** Mediation effects of serum albumin, uric acid, and GGT in the association between lifestyle OBS and pelvis BMD. **(C)** Mediation effects of serum albumin, uric acid, and GGT in the association between lifestyle OBS and total BMD. OBS: oxidative balance score; BMD: bone mineral density; GGT: gamma-glutamyl transpeptidase.

In the above analysis, significant differences were observed in serum albumin, GGT, and uric acid levels between the high and low lifestyle OBS groups. To further explore the potential mechanisms linking lifestyle OBS and BMD, we conducted mediation analyses using these three oxidative stress-related indicators as mediators. The results (Figures 3A–C) showed that lifestyle OBS was significantly associated with all three indicators (p<0.001), exhibiting a positive association with serum albumin and negative associations with GGT and uric acid. Regarding lumbar spine BMD, lifestyle OBS showed a significant direct effect (p = 0.001), but the mediation effects through serum albumin, uric acid, or GGT were not statistically significant (Figure 3A). For pelvic BMD, the direct effect of lifestyle OBS was also significant (p<0.001), and all three oxidative stress-related indicators were significantly associated with pelvis BMD (p<0.01, Figure 3B). Mediation analysis revealed that uric acid (mediation effect = −0.003, p<0.001) and GGT (mediation effect = −0.004, p = 0.004) had significant mediation effects, while the mediating effect of serum albumin was marginally significant (p = 0.052) (Figure 3B). For total BMD, a significant direct effect of lifestyle OBS was also observed (p<0.001) (Figure 3C). The mediation effects of uric acid (mediation effect = −0.001, p = 0.012) and GGT (mediation effect = −0.002, p<0.001) were statistically significant, whereas the mediation effect of serum albumin was not (p = 0.140). These findings suggest that GGT and uric acid may partially mediate the relationship between lifestyle OBS and BMD, particularly for pelvis and total BMD.

## 4 Discussion

This study investigated the relationship between OBS and BMD in adolescents. The results indicated that while total OBS and dietary OBS were not significantly associated with BMD, lifestyle OBS was positively correlated with lumbar spine, pelvic, and total BMD. However, after adjusting for confounding factors, the correlation between lifestyle OBS and BMD was reversed to negative. Further subgroup and interaction analyses revealed that the effect of lifestyle OBS on BMD was age-dependent, showing a positive correlation in younger individuals (≤13 years) and a negative correlation in older adolescents (>13 years). Additionally, a U-shaped non-linear relationship between lifestyle OBS and BMD was identified, with the inflection point approximating the median lifestyle OBS value.

Oxidative stress disrupts the balance between osteoclasts and osteoblasts, leading to chronic metabolic disorders. On the one hand, oxidative stress promotes the differentiation of pre-osteoclasts into osteoclasts and enhances bone resorption; on the other hand, elevated ROS levels induce osteocyte apoptosis, leading to an imbalance in bone remodeling and ultimately impairing bone formation (Zhivodernikov et al., 2023). Increasing research has explored the relationship between systemic oxidative stress status and BMD. For example, a recent study highlighted the association between OBS and lumbar spine BMD in adults aged 20–40 years, suggesting that adopting an antioxidant diet and lifestyle may help young individuals maintain bone mass (Tao et al., 2024). Another study observed a significant positive association between OBS and BMD in the lumbar spine and femur of postmenopausal women (Mahmoodi et al., 2025). Our study is the first to investigate the association between OBS and BMD in children and adolescents. Childhood and adolescence represent critical periods for bone development and maturation (Lopez-Gonzalez et al., 2021). Our findings align with previous studies examining the impact of lifestyle factors on bone health. Multiple studies have demonstrated that PA positively influences BMD by stimulating bone formation and enhancing peak bone mass acquisition (Han et al., 2021; Krahenbuhl et al., 2018). Our results also indicate a significant association between lifestyle OBS and BMD. Based on this, we further explored the potential mediating role of oxidative stress in the relationship between lifestyle OBS and BMD. The results indicated that GGT and uric acid exhibited significant mediation effects for pelvic and total BMD, suggesting that they may partially mediate the impact of lifestyle OBS on BMD. Previous studies have also reported potential negative associations between OBS and both uric acid and GGT (Cho et al., 2018; Yang Y. et al., 2024), further supporting the plausibility of oxidative stress as a key mediating factor from a biological mechanism perspective. Oxidative stress regulates several critical signaling pathways in bone cells. For example, the Wnt/β-catenin pathway plays a central role in osteoblast differentiation and bone formation (Chen et al., 2019; Shen et al., 2022). Prior research has shown that the ROS/Wnt/β-catenin axis is associated with osteogenesis (Wang and Liu, 2025). In addition, oxidative stress may influence the proliferation and apoptosis of bone cells via the NF-κB and MAPK signaling pathways, thereby disrupting the balance of bone remodeling (Liu et al., 2023; Thummuri et al., 2017). Our mediation analysis further validates the pivotal role of oxidative stress in the relationship between lifestyle OBS and BMD.

Interestingly, the initially observed significant positive correlation between lifestyle OBS and BMD was reversed to negative after adjusting for confounding factors. This contrasts with previous research, which generally suggests that PA has a sustained positive effect on BMD (Han et al., 2021; Ondrak and Morgan). Sensitivity analysis suggested that age might be a key modulating factor in the relationship between lifestyle OBS and BMD. Further interaction analysis confirmed that age was an interactive factor affecting this relationship. Previous studies have shown that oxidative stress accelerates age-related bone loss (Qiao et al., 2020). In this study, lifestyle OBS played a crucial role in increasing BMD primarily in the younger age group. Subgroup analysis revealed that lifestyle OBS had a positive impact on BMD in adolescents aged ≤13 years, whereas it was negatively associated with BMD in adolescents >13 years. This discrepancy may be attributed to differences in bone development stages and responses to mechanical stress. Younger individuals exhibit greater bone plasticity and responsiveness to low mechanical loads, whereas older adolescents require higher mechanical loads to elicit a bone response (Kopiczko et al., 2020). Additionally, the positive correlation between BMI and BMD may have a saturation effect. Among adolescents, the BMI threshold for BMD saturation may change significantly with age (Ouyang et al., 2022). We propose that the determinants of bone health shift across different developmental stages. In childhood and early adolescence, modifiable lifestyle factors such as PA play a dominant role in bone mass accumulation, whereas in late adolescence, intrinsic factors such as hormonal regulation, skeletal maturation, and genetic predisposition may have a greater impact. Therefore, interventions aimed at improving lifestyle OBS should be tailored to different age groups to maximize their effectiveness in promoting bone health.

In our study, another noteworthy finding was the U-shaped relationship between lifestyle OBS and BMD, suggesting that the beneficial effects of lifestyle OBS on BMD may be dose-dependent, becoming evident only after exceeding a certain threshold. We observed that the inflection point for lifestyle OBS was around 5, which was also its median value, indicating that lower levels of lifestyle OBS may not be sufficient to confer bone health benefits. This suggests that merely adopting partial healthy behaviors may not effectively enhance BMD; rather, a comprehensive and multidimensional intervention approach is necessary to optimize bone health. Additionally, this dose-response pattern has also been observed in previous studies examining the relationship between PA and BMD, where PA was found to exhibit a J-shaped association with BMD (Yang H. et al., 2024). Notably, lifestyle OBS may influence BMD by modulating oxidative stress levels. Previous research has demonstrated that a healthy lifestyle, such as regular physical activity, can reduce oxidative stress and thereby lower the risk of bone loss (Faienza et al., 2020). Our results indicate that individuals with lifestyle OBS >5 engaged in significantly more weekly PA. We speculate that the U-shaped relationship between lifestyle OBS and BMD may be related to a “threshold effect” of oxidative stress. Specifically, when OBS is low, the antioxidant capacity may be insufficient to counteract endogenous ROS, thereby impairing bone metabolism. In addition, other factors may also jointly regulate the relationship between OBS and BMD. For example, tobacco exposure also affects bone metabolism. Prior studies have shown that cotinine inhibits catalase and glutathione reductase activity, leading to impaired osteoblast function (Aspera-Werz et al., 2018). We found that individuals with lifestyle OBS >5 had significantly lower cotinine levels compared to those with lifestyle OBS ≤5. These findings further underscore the necessity of promoting healthy lifestyles to mitigate oxidative stress and optimize bone metabolism.

Furthermore, our study identified significant gender differences in several OBS-related variables. Compared to females, males exhibited higher levels of PA and cotinine, which aligns with previous epidemiological studies showing that adolescent boys engage in more physical exercise and have higher tobacco exposure levels (Rosselli et al., 2020; Liu et al., 2025). Interestingly, despite these differences, males had significantly lower lumbar spine BMD than females. This is consistent with prior findings indicating that, due to differences in endogenous sex hormone levels, males reach their peak bone mass later than females (Ausili et al., 2012; Xu et al., 2022). Furthermore, our study identified significant gender differences in several OBS-related variables. Compared to females, males exhibited higher levels of PA and cotinine, which aligns with previous epidemiological studies showing that adolescent boys engage in more physical exercise and have higher tobacco exposure levels (Yang Y. et al., 2024; Chen et al., 2019). Interestingly, despite these differences, males had significantly lower lumbar spine BMD than females. This is consistent with prior findings indicating that, due to differences in endogenous sex hormone levels, males reach their peak bone mass later than females (Shen et al., 2022; Wang and Liu, 2025). Further analysis revealed that the interaction between lifestyle OBS and gender was not statistically significant for lumbar spine, pelvic, or total BMD, suggesting that the effect of lifestyle OBS on BMD is relatively consistent across genders. This phenomenon may be partly explained by the fact that, although males exhibit higher levels of physical activity and cotinine, these factors might offset each other within the composite lifestyle OBS score. Moreover, since lifestyle OBS incorporates both pro-oxidants and antioxidants into a single integrated index, it may obscure the influence of individual gender-related differences in specific variables (Hernandez-Ruiz et al., 2022). Therefore, despite the presence of potential physiological and behavioral differences between genders, lifestyle OBS demonstrated strong stability and consistency in its association with BMD. Nevertheless, gender-specific physiological mechanisms—such as differences in sex hormone levels, muscle mass, and skeletal loading—as well as sociocultural behavior patterns, remain important determinants of bone health (Cauley, 2015; Mattia et al., 2023). Future studies should incorporate longitudinal data and hormonal assessments to further elucidate the potential moderating role of gender in the relationship between OBS and BMD.

Despite these valuable insights, our study has several limitations. First, the cross-sectional nature of our analysis prevents us from inferring causality between lifestyle OBS and BMD. Second, while our study controlled for major confounders such as age and gender, residual confounding from unmeasured factors (e.g., genetic predisposition) cannot be entirely ruled out. Third, a substantial number of participants were excluded due to missing data on OBS components, which may introduce selection bias and affect the generalizability of our findings. Fourth, although NHANES data are designed to be nationally representative through a complex sampling strategy, we did not apply sampling weights in our analysis. This decision was made to focus on internal associations and improve model efficiency, but it may limit the national representativeness of our findings. Lastly, the current OBS scoring system assumes equal weighting for each component. This approach does not account for the differential contributions of individual pro-oxidants and antioxidants, nor does it reflect possible synergistic or antagonistic effects between components. Although this method is widely used in epidemiological research due to its simplicity and comparability, it may introduce bias and limit the accuracy of oxidative balance estimation. Therefore, future studies should consider developing a weighted OBS scoring system to further enhance the accuracy of OBS estimation. In addition, prospective follow-up studies are recommended to better clarify the causal relationship between lifestyle OBS and BMD, and to evaluate the long-term impact of different OBS levels on bone changes, particularly the underlying mechanisms during the period of rapid bone mass accrual in adolescence. This would be of great significance for formulating early intervention strategies and optimizing bone health in adolescents.

## 5 Conclusion

In conclusion, our study provides novel insights into the complex relationship between adolescent lifestyle OBS and BMD, revealing the critical role of age in this association. These findings emphasize the importance of considering developmental stages when designing lifestyle interventions for bone health. Future research should focus on longitudinal evaluations to clarify the long-term effects of lifestyle OBS on bone outcomes and explore the underlying mechanisms mediating these associations.

## Data Availability

The raw data supporting the conclusions of this article will be made available by the authors, without undue reservation.

## Supplementary material

SUPPLEMENTARY FIGURE S1 Flow chart of included study population. NHANES: National Health and Nutrition Examination Survey, OBS: oxidative balance score; BMD: bone mineral density.

## References

[B1] Ahn M. B. Yoo I. H. (2023). Risk factors of low bone mineral density in newly diagnosed pediatric inflammatory bowel disease. Nutrients 15 (24), 5048. 10.3390/nu15245048 38140307 PMC10746078

[B2] Aspera-Werz R. H. Ehnert S. Heid D. Zhu S. Chen T. Braun B. (2018). Nicotine and cotinine inhibit catalase and glutathione reductase activity contributing to the impaired osteogenesis of SCP-1 cells exposed to cigarette smoke. Oxid. Med. Cell Longev. 2018, 3172480. 10.1155/2018/3172480 30533170 PMC6250005

[B3] Ausili E. Rigante D. Salvaggio E. Focarelli B. Rendeli C. Ansuini V. (2012). Determinants of bone mineral density, bone mineral content, and body composition in a cohort of healthy children: influence of sex, age, puberty, and physical activity. Rheumatol. Int. 32 (9), 2737–2743. 10.1007/s00296-011-2059-8 21809005

[B4] Cauley J. A. (2015). Estrogen and bone health in men and women. Steroids 99 (Pt A), 11–15. 10.1016/j.steroids.2014.12.010 25555470

[B5] Chang Y. Yu C. Dai X. Sun H. Tang T. (2024). Association of dietary inflammatory index and dietary oxidative balance score with gastrointestinal cancers in NHANES 2005-2018. BMC Public Health 24 (1), 2760. 10.1186/s12889-024-20268-4 39385181 PMC11465896

[B6] Chen K. Li S. Xie Z. Liu Y. Li Y. Mai J. (2024). Association between oxidative balance score, systemic inflammatory response index, and cardiovascular disease risk: a cross-sectional analysis based on NHANES 2007-2018 data. Front. Nutr. 11, 1374992. 10.3389/fnut.2024.1374992 38899319 PMC11186475

[B7] Chen X. J. Shen Y. S. He M. C. Yang F. Yang P. Pang F. X. (2019). Polydatin promotes the osteogenic differentiation of human bone mesenchymal stem cells by activating the BMP2-Wnt/β-catenin signaling pathway. Biomed. Pharmacother. 112, 108746. 10.1016/j.biopha.2019.108746 30970530

[B8] Cho A. R. Kwon Y. J. Lim H. J. Lee H. S. Kim S. Shim J. Y. (2018). Oxidative balance score and serum gamma-glutamyltransferase level among Korean adults: a nationwide population-based study. Eur. J. Nutr. 57 (3), 1237–1244. 10.1007/s00394-017-1407-1 28258305

[B9] Deng K. L. Yang W. Y. Hou J. L. Li H. Feng H. Xiao S. M. (2021). Association between body composition and bone mineral density in children and adolescents: a systematic review and meta-analysis. Int. J. Environ. Res. Public Health 18 (22), 12126. 10.3390/ijerph182212126 34831882 PMC8618958

[B10] Faienza M. F. Lassandro G. Chiarito M. Valente F. Ciaccia L. Giordano P. (2020). How physical activity across the lifespan can reduce the impact of bone ageing: a literature review. Int. J. Environ. Res. Public Health 17 (6), 1862. 10.3390/ijerph17061862 32183049 PMC7143872

[B11] Fu Y. Wang G. Liu J. Li M. Dong M. Zhang C. (2022). Stimulant use and bone health in US children and adolescents: analysis of the NHANES data. Eur. J. Pediatr. 181 (4), 1633–1642. 10.1007/s00431-021-04356-w 35091797

[B12] Gusmao M. B. F. Oliveira V. V. Santos N. Melo L. C. (2023). Assessing bone mineral density in children and adolescents living with HIV and on treatment with tenofovir disoproxil fumarate: a systematic review. Rev. Paul. Pediatr. 42, e2023042. 10.1590/1984-0462/2024/42/2023042 37971172 PMC10637732

[B13] Han C. S. Kim H. K. Kim S. (2021). Effects of adolescents' lifestyle habits and body composition on bone mineral density. Int. J. Environ. Res. Public Health 18 (11), 6170. 10.3390/ijerph18116170 34200352 PMC8201294

[B14] Hasani M. Alinia S. P. Khazdouz M. Sobhani S. Mardi P. Ejtahed H. S. (2023). Oxidative balance score and risk of cancer: a systematic review and meta-analysis of observational studies. BMC Cancer 23 (1), 1143. 10.1186/s12885-023-11657-w 38001409 PMC10675899

[B15] Hernandez-Ruiz A. Garcia-Villanova B. Guerra-Hernandez E. J. Carrion-Garcia C. J. Amiano P. Sanchez M. J. (2022). Oxidative balance scores (OBSs) integrating nutrient, food and lifestyle dimensions: development of the NutrientL-OBS and FoodL-OBS. Antioxidants (Basel) 11 (2), 300. 10.3390/antiox11020300 35204183 PMC8868253

[B16] Kopiczko A. Adamczyk J. G. Lopuszanska-Dawid M. (2020). Bone mineral density in adolescent boys: cross-sectional observational study. Int. J. Environ. Res. Public Health 18 (1), 245. 10.3390/ijerph18010245 33396391 PMC7795160

[B17] Krahenbuhl T. Guimaraes R. F. Barros Filho A. A. Goncalves E. M. (2018). Bone geometry and physical activity in children and adolescents: systematic review. Rev. Paul. Pediatr. 36 (2), 230–237. 10.1590/1984-0462/;2018;36;2;00005 29412432 PMC6038793

[B18] Liu F. You F. Yang L. Du X. Li C. Chen G. (2024). Nonlinear relationship between oxidative balance score and hyperuricemia: analyses of NHANES 2007-2018. Nutr. J. 23 (1), 48. 10.1186/s12937-024-00953-1 38704549 PMC11069158

[B19] Liu Y. Li L. Chen Z. Ren S. He R. Liang Y. (2025). Relationship between parental smoking and adolescent smoking: gender differences and mediation of resilience. BMC Public Health 25 (1), 434. 10.1186/s12889-025-21457-5 39901135 PMC11792306

[B20] Liu Z. Yao X. Jiang W. Zhou Z. Yang M. (2023). Sodium butyrate enhances titanium nail osseointegration in ovariectomized rats by inhibiting the PKCα/NOX4/ROS/NF-κB pathways. J. Orthop. Surg. Res. 18 (1), 556. 10.1186/s13018-023-04013-y 37528483 PMC10394859

[B21] Lopez-Gonzalez D. Wells J. C. Cortina-Borja M. Fewtrell M. Partida-Gaytan A. Clark P. (2021). Reference values for bone mineral density in healthy Mexican children and adolescents. Bone 142, 115734. 10.1016/j.bone.2020.115734 33166709

[B22] Mahmoodi M. Souni F. Shateri Z. Hosseini A. S. Nouri M. Ghadiri M. (2025). The association of phytochemical index and oxidative balance score with bone mineral density: a case-control study. J. Health Popul. Nutr. 44 (1), 40. 10.1186/s41043-025-00747-z 39948660 PMC11827369

[B23] Mattia L. Gossiel F. Walsh J. S. Eastell R. (2023). Effect of age and gender on serum growth differentiation factor 15 and its relationship to bone density and bone turnover. Bone Rep. 18, 101676. 10.1016/j.bonr.2023.101676 37090856 PMC10113774

[B24] Ondrak K. S. Morgan D. W. (2007). Physical activity, calcium intake and bone health in children and adolescents. Sports Med. 37 (7), 587–600. 10.2165/00007256-200737070-00003 17595154

[B25] Ouyang Y. Quan Y. Guo C. Xie S. Liu C. Huang X. (2022). Saturation effect of body mass index on bone mineral density in adolescents of different ages: a population-based study. Front. Endocrinol. (Lausanne) 13, 922903. 10.3389/fendo.2022.922903 35865310 PMC9294630

[B26] Qiao W. Yu S. Sun H. Chen L. Wang R. Wu X. (2020). 1,25-Dihydroxyvitamin D insufficiency accelerates age-related bone loss by increasing oxidative stress and cell senescence. Am. J. Transl. Res. 12 (2), 507–518. 32194899 PMC7061846

[B27] Rosselli M. Ermini E. Tosi B. Boddi M. Stefani L. Toncelli L. (2020). Gender differences in barriers to physical activity among adolescents. Nutr. Metab. Cardiovasc Dis. 30 (9), 1582–1589. 10.1016/j.numecd.2020.05.005 32605880

[B28] Shen M. Wang L. Feng L. Gao Y. Li S. Wu Y. (2022). bFGF-Loaded mesoporous silica nanoparticles promote bone regeneration through the Wnt/β-Catenin signalling pathway. Int. J. Nanomedicine 17, 2593–2608. 10.2147/IJN.S366926 35698561 PMC9188412

[B29] Song L. Li H. Fu X. Cen M. Wu J. (2023). Association of the oxidative balance score and cognitive function and the mediating role of oxidative stress: evidence from the national health and nutrition examination survey (NHANES) 2011-2014. J. Nutr. 153 (7), 1974–1983. 10.1016/j.tjnut.2023.05.014 37187352

[B30] Song L. Zhou H. Yang Q. He N. Fu F. Li W. (2024). Association between the oxidative balance score and thyroid function: results from the NHANES 2007-2012 and Mendelian randomization study. PLoS One 19 (3), e0298860. 10.1371/journal.pone.0298860 38498431 PMC10947682

[B31] Tang Y. Peng B. Liu J. Liu Z. Xia Y. Geng B. (2022). Systemic immune-inflammation index and bone mineral density in postmenopausal women: a cross-sectional study of the national health and nutrition examination survey (NHANES) 2007-2018. Front. Immunol. 13, 975400. 10.3389/fimmu.2022.975400 36159805 PMC9493473

[B32] Tao Y. A. Long L. Gu J. X. Wang P. Y. Li X. Li X. L. (2024). Associations of oxidative balance score with lumbar spine osteopenia in 20-40 years adults: NHANES 2011-2018. Eur. Spine J. 33 (9), 3343–3351. 10.1007/s00586-024-08424-1 39168893

[B33] Tao Z. S. Ma T. (2024). Sodium butyrate protect bone mass in lipopolysaccharide-treated rats by reducing oxidative stress and inflammatory. Redox Rep. 29 (1), 2398891. 10.1080/13510002.2024.2398891 39284587 PMC11407388

[B34] Thummuri D. Naidu V. G. M. Chaudhari P. (2017). Carnosic acid attenuates RANKL-Induced oxidative stress and osteoclastogenesis *via* induction of Nrf2 and suppression of NF-κB and MAPK signalling. J. Mol. Med. Berl. 95 (10), 1065–1076. 10.1007/s00109-017-1553-1 28674855

[B35] Tian X. Xue B. Wang B. Lei R. Shan X. Niu J. (2022). Physical activity reduces the role of blood cadmium on depression: a cross-sectional analysis with NHANES data. Environ. Pollut. 304, 119211. 10.1016/j.envpol.2022.119211 35341822

[B36] Wang X. Hu J. Liu L. Zhang Y. Dang K. Cheng L. (2023). Association of dietary inflammatory index and dietary oxidative balance score with all-cause and disease-specific mortality: findings of 2003-2014 national health and nutrition examination survey. Nutrients 15 (14), 3148. 10.3390/nu15143148 37513566 PMC10383761

[B37] Wang Y. N. Liu S. (2025). Lipid droplet accumulation impairs osseointegration by disturbing the osteogenesis-osteoclasis balance on titanium implant surface in hyperlipidemia. BMC Oral Health 25 (1), 823. 10.1186/s12903-025-06218-5 40437476 PMC12117928

[B38] Wu C. Ren C. Song Y. Gao H. Pang X. Zhang L. (2023). Gender-specific effects of oxidative balance score on the prevalence of diabetes in the US population from NHANES. Front. Endocrinol. (Lausanne) 14, 1148417. 10.3389/fendo.2023.1148417 37214249 PMC10194026

[B39] Xu K. Fu Y. Cao B. Zhao M. (2022). Association of sex hormones and sex hormone-binding globulin levels with bone mineral density in adolescents aged 12-19 years. Front. Endocrinol. (Lausanne) 13, 891217. 10.3389/fendo.2022.891217 35669686 PMC9163352

[B40] Xu W. Mu D. Wang Y. Wang Y. Wang C. Zhang X. (2024b). Association between oxidative balance score and sarcopenia in US adults: NHANES 2011-2018. Front. Nutr. 11, 1342113. 10.3389/fnut.2024.1342113 38721026 PMC11076835

[B41] Xu Z. Liu D. Zhai Y. Tang Y. Jiang L. Li L. (2024a). Association between the oxidative balance score and all-cause and cardiovascular mortality in patients with diabetes and prediabetes. Redox Biol. 76, 103327. 10.1016/j.redox.2024.103327 39186882 PMC11389538

[B42] Yang H. Li B. Li H. Zhou M. Li B. Guo J. (2024b). The independent and joint association between physical activity, sleep duration and daily sitting time with bone mineral density: a real world study from NHANES 2007-2018. Bone 189, 117264. 10.1016/j.bone.2024.117264 39332788

[B43] Yang Y. Wu Z. An Z. Li S. (2024a). Association between oxidative balance score and serum uric acid and hyperuricemia: a population-based study from the NHANES (2011-2018). Front. Endocrinol. (Lausanne) 15, 1414075. 10.3389/fendo.2024.1414075 38966221 PMC11222604

[B44] Yuan K. Xie X. Huang W. Li D. Zhao Y. Yang H. (2024). Elucidating causal relationships of diet-derived circulating antioxidants and the risk of osteoporosis: a Mendelian randomization study. Front. Genet. 15, 1346367. 10.3389/fgene.2024.1346367 38911297 PMC11190308

[B45] Zhang C. Li H. Li J. Hu J. Yang K. Tao L. (2023). Oxidative stress: a common pathological state in a high-risk population for osteoporosis. Biomed. Pharmacother. 163, 114834. 10.1016/j.biopha.2023.114834 37163779

[B46] Zhang W. Peng S. F. Chen L. Chen H. M. Cheng X. E. Tang Y. H. (2022). Association between the oxidative balance score and telomere length from the national health and nutrition examination survey 1999-2002. Oxid. Med. Cell Longev. 2022, 1345071. 10.1155/2022/1345071 35186180 PMC8850082

[B47] Zhivodernikov I. V. Kirichenko T. V. Markina Y. V. Postnov A. Y. Markin A. M. (2023). Molecular and cellular mechanisms of osteoporosis. Int. J. Mol. Sci. 24 (21), 15772. 10.3390/ijms242115772 37958752 PMC10648156

[B48] Zhou Z. Han Y. (2024). Association between oxidative balance score and hearing loss: a cross-sectional study from the NHANES database. Front. Nutr. 11, 1375545. 10.3389/fnut.2024.1375545 38812938 PMC11135173

[B49] Zhu H. Jin L. Zhang Z. Lu C. Jiang Q. Mou Y. (2025). Oxidative balance scores and gallstone disease: mediating effects of oxidative stress. Nutr. J. 24 (1), 4. 10.1186/s12937-025-01073-0 39789597 PMC11720334

