# Supplementary material for: Age-dependent association between lifestyle oxidative balance score and bone mineral density in children and adolescents: evidence from the NHANES 2015–2018
Source: Front Physiol. 2025 Jul 4;16:1618996. doi: 10.3389/fphys.2025.1618996 (PMC12271102; doi:10.3389/fphys.2025.1618996)
Supplement: Supplementary file 1 [file Table1.docx]

**Table S1 OBS components**

| OBS components | Property | Male | | | Female | | |
| --- | --- | --- | --- | --- | --- | --- | --- |
|  |  | 0 | 1 | 2 | 0 | 1 | 2 |
| Dietary OBS |  |  |  |  |  |  |  |
| Dietary fiber (g/d) | A | Tertile 1 | Tertile 2 | Tertile 3 | Tertile 1 | Tertile 2 | Tertile 3 |
| Carotene (RE/d) | A | Tertile 1 | Tertile 2 | Tertile 3 | Tertile 1 | Tertile 2 | Tertile 3 |
| Riboflavin (mg/d) | A | Tertile 1 | Tertile 2 | Tertile 3 | Tertile 1 | Tertile 2 | Tertile 3 |
| Niacin (mg/d) | A | Tertile 1 | Tertile 2 | Tertile 3 | Tertile 1 | Tertile 2 | Tertile 3 |
| Vitamin B6 (mg/d) | A | Tertile 1 | Tertile 2 | Tertile 3 | Tertile 1 | Tertile 2 | Tertile 3 |
| Total folate (mcg/d) | A | Tertile 1 | Tertile 2 | Tertile 3 | Tertile 1 | Tertile 2 | Tertile 3 |
| Vitamin B12 (mcg/d) | A | Tertile 1 | Tertile 2 | Tertile 3 | Tertile 1 | Tertile 2 | Tertile 3 |
| Vitamin C (mg/d) | A | Tertile 1 | Tertile 2 | Tertile 3 | Tertile 1 | Tertile 2 | Tertile 3 |
| Vitamin E (ATE) (mg/d) | A | Tertile 1 | Tertile 2 | Tertile 3 | Tertile 1 | Tertile 2 | Tertile 3 |
| Calcium (mg/d) | A | Tertile 1 | Tertile 2 | Tertile 3 | Tertile 1 | Tertile 2 | Tertile 3 |
| Magnesium (mg/d) | A | Tertile 1 | Tertile 2 | Tertile 3 | Tertile 1 | Tertile 2 | Tertile 3 |
| Zinc (mg/d) | A | Tertile 1 | Tertile 2 | Tertile 3 | Tertile 1 | Tertile 2 | Tertile 3 |
| Copper (mg/d) | A | Tertile 1 | Tertile 2 | Tertile 3 | Tertile 1 | Tertile 2 | Tertile 3 |
| Selenium (mcg/d) | A | Tertile 1 | Tertile 2 | Tertile 3 | Tertile 1 | Tertile 2 | Tertile 3 |
| Total fat (g/d) | P | Tertile 3 | Tertile 2 | Tertile 1 | Tertile 3 | Tertile 2 | Tertile 1 |
| Iron (mg/d) | P | Tertile 3 | Tertile 2 | Tertile 1 | Tertile 3 | Tertile 2 | Tertile 1 |
| Lifestyle OBS |  |  |  |  |  |  |  |
| PA (MET, minute/week) | A | Tertile 1 | Tertile 2 | Tertile 3 | Tertile 1 | Tertile 2 | Tertile 3 |
| Alcohol (g/d) | P | ≥30 | 0-30 | no | ≥15 | 0-15 | no |
| BMI (kg/m^2^) | P | Tertile 3 | Tertile 2 | Tertile 1 | Tertile 3 | Tertile 2 | Tertile 1 |
| Cotinine (ng/mL) | P | Tertile 3 | Tertile 2 | Tertile 1 | Tertile 3 | Tertile 2 | Tertile 1 |

OBS: oxidative balance score; BMI: body mass index; PA: physical activity; MET: metabolic equivalent tasks; RE: retinol equivalent; ATE: alpha-tocopherol equivalent.
