# Supplementary material for: Age-dependent association between lifestyle oxidative balance score and bone mineral density in children and adolescents: evidence from the NHANES 2015–2018
Source: Front Physiol. 2025 Jul 4;16:1618996. doi: 10.3389/fphys.2025.1618996 (PMC12271102; doi:10.3389/fphys.2025.1618996)
Supplement: Supplementary file 2 [file Table2.docx]

**Table S2 Association of lifestyle OBS with BMD based on threshold grouping**

|  | Lifestyle OBS level | β (95%CI) | p | LRT-p |
| --- | --- | --- | --- | --- |
| Lumbar spine BMD | <4.559 | -0.043 (-0.060, -0.025) | <0.001 | <0.001 |
|  | ≥4.559 | 0.066 (0.051, 0.080) | <0.001 |  |
| Pelvis BMD | <4.712 | -0.057 (-0.077, -0.037) | <0.001 | <0.001 |
|  | ≥4.712 | 0.074 (0.055, 0.092) | <0.001 |  |
| Total BMD | <4.681 | -0.035 (-0.049, -0.022) | <0.001 | <0.001 |
|  | ≥4.681 | 0.059 (0.046, 0.070) | <0.001 |  |

LRT-p<0.05 indicates a non-linear relationship.

OBS: oxidative balance score; BMD: bone mineral density; CI: confidence interval; LRT: log-likelihood ratio.
